# Variation in the X:Autosome Distribution of Male-Biased Genes among *Drosophila melanogaster* Tissues and Its Relationship with Dosage Compensation

**DOI:** 10.1093/gbe/evv117

**Published:** 2015-06-24

**Authors:** Ann Kathrin Huylmans, John Parsch

**Affiliations:** Faculty of Biology, Ludwig Maximilian University of Munich, Planegg, Germany

**Keywords:** transcriptome, brain, sexual dimorphism, sex chromosomes

## Abstract

Genes that are expressed differently between males and females (sex-biased genes) often show a nonrandom distribution in their genomic location, particularly with respect to the autosomes and the X chromosome. Previous studies of *Drosophila melanogaster* found a general paucity of male-biased genes on the X chromosome, although this is mainly limited to comparisons of whole flies or body segments containing the reproductive organs. To better understand the chromosomal distribution of sex-biased genes in various tissues, we used a common analysis framework to analyze microarray and RNA sequence data comparing male and female gene expression in individual tissues (brain, Malpighian tubule, and gonads), composite structures (head and gonadectomized carcass), and whole flies. Although there are relatively few sex-biased genes in the brain, there is a strong and highly significant enrichment of male-biased genes on the X chromosome. A weaker enrichment of X-linked male-biased genes is seen in the head, suggesting that most of this signal comes from the brain. In all other tissues, there is either no departure from the random expectation or a significant paucity of male-biased genes on the X chromosome. The brain and head also differ from other tissues in that their male-biased genes are significantly closer to binding sites of the dosage compensation complex. We propose that the interplay of dosage compensation and sex-specific regulation can explain the observed differences between tissues and reconcile disparate results reported in previous studies.

## Introduction

Recent genomic studies have shown that sex chromosomes differ from autosomes in their gene content and expression (reviewed by Ellegren and Parsch 2007; Parsch and Ellegren 2013). Initial transcriptomic studies of the model eukaryote *Drosophila melanogaster* revealed that there is a significant paucity of genes with male-biased expression (male-biased genes, MBG) and a slight excess of genes with female-biased expression (female-biased genes, FBG) on the X chromosome relative to the autosomes (Parisi et al 2003; Ranz et al. 2003). These phenomena have been termed “demasculinization” and “feminization” of the X chromosome, respectively. More recent studies, however, have found that demasculinization of the X chromosome is not observed in all body parts or tissues. For example, Meisel et al. (2012) did not find a general paucity of X-linked MBG when comparing adult head, thorax, or whole larvae between males and females of four *Drosophila* species. To the contrary, they observed a slight excess of MBG in the heads of *D*. *melanogaster* and *Drosophila mojavensis* (Meisel et al. 2012). Two other recent studies reported a significant enrichment of X-linked MBG in the head and brain of *D*. *melanogaster* (Chang et al. 2011; Catalán et al. 2012).

Several hypotheses have been put forth to explain the observed differences in sex-biased gene content between the X chromosome and the autosomes. One possible explanation is sexual antagonism, in which there is conflict between the sexes regarding the optimum level of gene expression (Ellegren and Parsch 2007; Parsch and Ellegren 2013). Under such a scenario, the fate of an allele that influences the expression of a sexually antagonistic gene will differ depending on its dominance and whether it is X-linked or autosomal (Rice 1984; Charlesworth et al. 1987). In general, the X chromosome is expected to be a hotspot for sexually antagonistic alleles, favoring the accumulation of recessive male-beneficial alleles and dominant female-beneficial alleles. There is experimental evidence that genes with sexually antagonistic expression are enriched on the *D*. *melanogaster* X chromosome (Innocenti and Morrow 2010). However, there is not a clear link between sex-biased expression and sexual antagonism (Innocenti and Morrow 2010; Parsch and Ellegren 2013). Furthermore, the type of sexually antagonistic alleles that are expected to accumulate on the X chromosome depends on key parameters, such as their degree of dominance and the magnitude of their effect on fitness in the two sexes (Fry 2010), which are typically unknown.

Another factor that could influence the genomic distribution of sex-biased genes is a difference in gene content between the X chromosome and the autosomes. For example, the X chromosome is enriched with “young” genes (i.e., those that are present in only a restricted taxonomic group, including retrogenes and de novo genes) and these genes often show sex-biased expression (Betrán et al. 2002; Levine et al. 2006; Zhang et al. 2010; Palmieri et al. 2014). The X chromosome is also depauperate in genes with tissue-specific expression (Mikhaylova and Nurminsky 2011; Meisel et al. 2012; although see Vibranovski et al. 2012). In particular, MBG expressed in testis tend to have highly tissue-specific expression (Mikhaylova and Nurminsky 2011; Meisel et al. 2012), which could explain their paucity on the X chromosome. Genes expressed in the male accessory gland also show high tissue-specificity, although this alone cannot account for their underrepresentation on the X chromosome (Meisel et al. 2012).

The distribution of sex-biased genes on the X chromosome and autosomes could also be influenced by regulatory mechanisms specific to the X chromosome. For example, it has been proposed that in *Drosophila* the X chromosome is transcriptionally silenced in the male germline through a process analogous to the meiotic sex chromosome inactivation (MSCI) that occurs in mammals (Lifschytz and Lindsley 1972; Betrán et al. 2002; Vibranovski et al. 2009). Although there has been debate regarding the extent of MSCI in *Drosophila* and whether or not it is limited to meiosis (Meiklejohn et al. 2011; Mikhaylova and Nurminsky 2011, 2012; Vibranovski et al. 2012), experimental studies have shown that the expression of testis-specific reporter genes is greatly suppressed when they are X-linked (Hense et al. 2007; Kemkemer et al. 2011, 2014; Meiklejohn et al. 2011). This indicates that there is a mechanism, possibly distinct from MSCI, that limits the expression of X-linked genes in testis. However, the current data and experimental approaches are not able to determine the nature of this mechanism and more studies are needed (Vibranovski 2014).

Another regulatory mechanism that may influence sex-biased expression on the X chromosome is dosage compensation, which in *Drosophila* occurs through the upregulation of the male X chromosome (reviewed by Straub et al. 2005). Thus, in the absence of dosage compensation, one would expect to see an underrepresentation of MBG on the X chromosome. Although dosage compensation appears to be ubiquitous in somatic tissues, there is evidence that it does not occur in the male germline (Meiklejohn et al. 2011; but see Gupta et al. 2006; Deng et al. 2011), which could explain the paucity of X-linked MBG seen when samples containing reproductive tissues are compared (Meiklejohn and Presgraves 2012). In the soma, it has been suggested that the mechanism of dosage compensation may influence the chromosomal distribution of sex-biased genes in two opposing ways. First, because the establishment of male-biased expression typically involves the upregulation of expression in males (Connallon and Knowles 2006; Vicoso and Charlesworth 2009) it may be constrained by the constitutive hypertranscription of the male X chromosome (Corona et al. 2002; Vicoso and Charlesworth 2009). Consistent with this interpretation, genes with male-biased expression in whole or gonadectomized flies tend to be located far away from the binding sites of the dosage compensation complex (DCC) (Bachtrog et al. 2010). Second, it has been proposed that genes that are close to DCC binding sites may be overcompensated, having their expression increased more than the expected 2-fold (Chang et al. 2011). Under this scenario, one would expect MBG to be located close to DCC binding sites, which has been observed for head and brain (Chang et al. 2011; Catalán et al. 2012).

To investigate the genomic distribution of sex-biased genes and its relationship with dosage compensation and other factors, we analyzed several *D*. *melanogaster* data sets that compared male and female expression in various samples, ranging from individual tissues to whole flies (table 1). Because the original data were generated using different methodologies and experimental designs, we took great care to standardize our analysis as much as possible. We find that the brain is unique in showing an extreme excess of X-linked MBG and that these genes tend to be located close to DCC binding sites. For other tissues and whole flies, there is either no enrichment or a significant paucity of X-linked MBG and they tend to be far away from DCC binding sites. These differences do not appear to be related to gene age or tissue-specific expression, but instead come from the interplay of dosage compensation and sex-specific regulation.

**Table 1:** Expression Data Sets Used in This Study

Data Set	Source	Method	Reference
1	Brain	RNA-seq	Catalán et al. (2012)
2	Head	RNA-seq	Chang et al. (2011)
3	Head	RNA-seq	Dalton et al. (2013)
4	Head	RNA-seq	Meisel et al. (2012)
5	Head	Microarray	Meisel et al. (2012)
6	Head	Microarray	Goldman and Arbeitman (2007)
7	Tubule	RNA-seq	Huylmans and Parsch (2014)
8	Whole fly	RNA-seq	Meisel et al. (2012)
9	Whole fly	Microarray	Parisi et al. (2004)
10	Whole fly	Meta-analysis	Gnad and Parsch (2006)
11	Gonadectomized	Microarray	Parisi et al. (2004)
12	Gonads	RNA-seq	Brown et al. (2014)
13	Gonads	RNA-seq	Gan et al. (2010)
14	Gonads	Microarray	Parisi et al. (2004)

## Materials and Methods

### Identification of Sex-Biased Genes

The expression data sets used in our analysis are listed in table 1. These include both RNA-seq and microarray data. Because the data were generated by different groups using different methodologies and experimental designs, it was necessary to standardize our analysis. For the RNA-seq data, we began with the raw sequences and applied a common pipeline for read mapping and statistical analysis. However, note that one of the RNA-seq data sets comes from a single somatic tissue (Malpighian tubule; data set 7) and was generated using the same fly strains, experimental procedures, and replication scheme used for the brain (data set 1). For each RNA-seq data set, the raw sequence reads were downloaded from the NCBI (*National Center for Biotechnology Information*) short read archive. The accession numbers are provided in supplementary table S1, Supplementary Material online. The reads were then mapped to the *D*. *melanogaster* transcriptome (FlyBase release 5.54) (St. Pierre et al. 2014), which included all protein-coding transcripts and noncoding RNAs. The mapping was done with NextGenMap (version 0.4.10) (Sedlazeck et al. 2013) using the default parameters.

The statistical detection of genes expressed differently between males and females was done with the Bioconductor (Gentleman et al. 2004) package DESeq2 (version 1.2.10) (Love et al. 2014) as implemented in R (version 3.0) (R Core Team 2014). This package was chosen because, in a previous study, it identified more differentially expressed genes than edgeR (version 3.6.8) (McCarthy et al. 2012) or baySeq (version 1.18.0) (Hardcastle and Kelly 2010) for one of the analyzed data sets (data set 7) (Huylmans and Parsch 2014). In cases where males and females of multiple strains or populations were compared, a two-factor analysis was carried out that accounted for both sex and strain (or population). The *P* values for differential expression were corrected for multiple testing using the method of Benjamini and Hochberg (1995). For all of the RNA-seq data sets, genes were considered sex-biased if their multiple-test-corrected *P* value was less than 5%.

To test whether the brain results (data set 1) were sensitive to the statistical method, we also used edgeR and baySeq to identify sex-biased genes in this tissue. Furthermore, we mapped the RNA-seq reads with Stampy (version 1.0.22) (Lunter and Goodson 2011) instead of NextGenMap to determine whether the results were sensitive to the mapping software. Although the numbers of sex-biased genes varied depending on the mapping software and the statistical method, the main results (a significant enrichment of MBG on the X chromosome; MBG significantly closer to DCC binding sites) were seen with all methods (supplementary table S2, Supplementary Material online). Similarly, we found that the brain results were not biased by genes with very low expression or with weak statistical support, as setting an expression threshold of RPKM (reads per kilobase per million mapped reads) > 1 or decreasing the false discovery rate (FDR) to 1% did not affect the above results or their statistical significance (supplementary table S2, Supplementary Material online).

For all but one of the microarray data sets, we used data from the Sebida database (Gnad and Parsch 2006). For the meta-analysis of whole fly expression (data set 10), the FDR was available for all genes and an FDR cutoff of 5% was used to define sex-biased genes. For the other microarray data sets, FDR estimates were not available and a nominal *P*-value cutoff of 0.05 was used to define sex-biased genes. For data set 14, the cutoff was increased to 0.10 in order to obtain a sufficient number of sex-biased genes. One additional head microarray data set (data set 5) (Meisel et al. 2012), which is not included in the Sebida database, was processed using the same methodology as the other microarray data sets in Sebida. For this data set, the software BAGEL (version 3.6) (Townsend and Hartl 2002) was used to determine the *P* value for differential expression between the sexes for each gene. A nominal *P* value of 0.01, corresponding to an FDR of 5% as determined by random permutations, was used to define significantly sex-biased genes. One other microarray study of sex-biased gene expression in the brain and central nervous system (Goldman and Arbeitman 2007) was excluded from the analysis, because it identified only four sex-biased genes.

The expected number of X-linked MBG (or FBG) for each data set was determined by multiplying the proportion of all genes in the data set that were X-linked by the total number of genes detected as male-biased (or female-biased) in that data set. For this, only genes that were expressed in the given data set (i.e., those that had enough RNA-seq reads for statistical analysis in DESeq2 or those that had no missing microarray data) were considered.

### Determination of the Distance between Genes and DCC Binding Sites

The locations of DCC binding sites on the X chromosome were taken from Alekseyenko et al. (2006) and Straub et al. (2013). The former used a ChIP-chip approach to identify binding sites of the DCC component MSL-3, whereas the latter used separate ChIP-seq experiments to identify binding sites of MLE, MSL-1, MSL-2, MSL-3, and MOF. All of the ChIP-chip and ChIP-seq experiments were performed on cultured S2 cells. Furthermore, we used the coordinates of HAS, which represent the initial entry point for DCC binding on the X chromosome, as defined by Straub et al. (2013) by the colocalization of MLE and MSL-2 binding sites. The distance analysis was carried out separately for each data set and DCC component. We calculated the distance between each X-linked gene and the nearest DCC binding site as the minimum distance in base pairs between the start (or end) of the DCC binding site and the start (or end) of the gene’s transcriptional unit. In cases where the DCC binding site overlapped with the transcriptional unit, the distance was set to zero. For each expression data set, only genes that were detected as expressed were taken into consideration when calculating the correlation between the male/female expression ratio and minimum DCC distance.

### Calculation of Tissue Specificity

For all genes in FlyBase release 5.54 (St. Pierre et al. 2014), the breadth of expression was calculated using the measurement τ (Yanai et al. 2005; Larracuente et al. 2008), which ranges from 0 (a broadly expressed gene) to 1 (a highly tissue-specific gene). The calculation of τ was done analogous to Meisel et al. (2012) and is based on 14 adult tissues from FlyAtlas (Chintapalli et al. 2007). Following the approach of Meisel (2011), the composite structures “head” and “carcass” were excluded and the expression of “spermatheca mated” and “spermatheca virgin” was averaged. For cases in which multiple array probes corresponded to the same gene, only the probe with the highest hybridization intensity was used.

### Estimation of Gene Age

Gene age was determined from the data of Zhang et al. (2010), which are based on orthology and synteny information across 12 completely sequenced *Drosophila* species (Clark et al. 2007). The genes were classified into age groups from 0 (*D*. *melanogaster*-specific) to 6 (emerged in or before the common ancestor of all 12 species) (supplementary fig. S6, Supplementary Material online).

## Results

### Genomic Distribution of Sex-Biased Genes

**Fig. 1.—:**
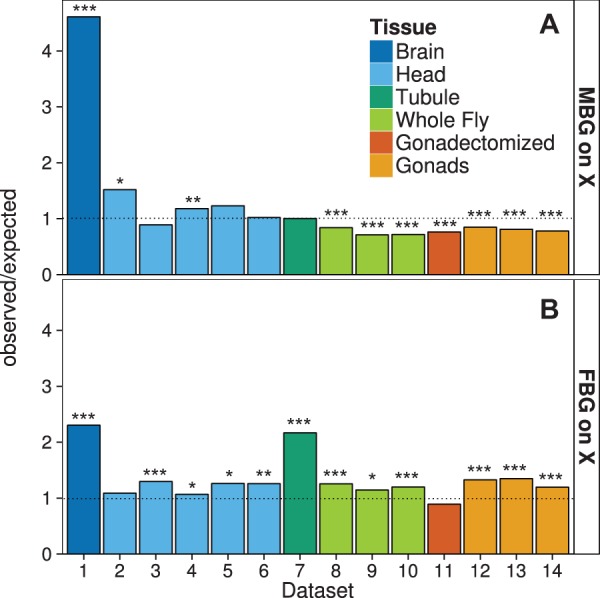
Relative abundance of sex-biased genes on the X chromosome. The ratio of observed to expected number of MBG (*A*) and FBG (*B*) on the X chromosome is shown. The data sets correspond to those listed in table 1. The expectation was determined from the proportion of all X-linked genes in each data set. Significance was determined by a Fisher exact test. *P < 0.05, **P < 0.01, ***P < 0.001.

To investigate the genomic distribution of sex-biased genes in various tissues and composite structures, we analyzed data from 14 different microarray and RNA sequencing (RNA-seq) experiments (table 1). The number of sex-biased genes varied greatly among data sets, with the highest numbers in whole flies or gonads and the lowest numbers in the brain and some head data sets (table 2). As can be seen in figure 1*A*, only the brain shows a very strong enrichment of MBG on the X chromosome, with over 75% (97 of 128) of the MBG being X-linked. A slight, but significant, enrichment of X-linked MBG is seen for two of the five head data sets. All other data sets show either no departure from the random expectation or a significant paucity of MBG on the X chromosome (fig. 1*A*). In particular, our analyses of whole flies and gonads confirm previous reports of demasculinization of the X chromosome in samples that include reproductive tissues (Parisi et al. 2003; Ranz et al. 2003; Meisel et al. 2012). We also see a general pattern of feminization of the X chromosome, which was significant in 12 of the 14 data sets, including the brain (fig. 1*B*).

**Table 2:** Numbers of Sex-Biased Genes in Each Data Set

Data Set	Source	MBG_A	MBG_X (%)	FBG_A	FBG_X (%)	UBG_A	UBG_X (%)
1	Brain	31	97 (76)	87	53 (38)	9,102	1,683 (16)
2	Head	87	31 (26)	194	45 (19)	6,619	1,367 (17)
3	Head	673	116 (15)	734	200 (21)	7,039	1,354 (16)
4	Head	1,519	368 (20)	1,350	289 (18)	5,062	912 (15)
5	Head	161	40 (20)	273	70 (21)	9,532	1,813 (16)
6	Head	688	133 (16)	658	164 (20)	6,496	1,182 (15)
7	Tubule	1,180	223 (16)	595	310 (34)	8,789	1,450 (14)
8	Whole fly	4,285	642 (13)	3,310	802 (20)	4,106	707 (15)
9	Whole fly	1,936	268 (12)	1,494	364 (20)	3,807	862 (19)
10	Whole fly	2,490	324 (12)	3,275	781 (19)	5,021	957 (16)
11	Gonadectomized	565	87 (13)	537	99 (16)	4,910	1,086 (18)
12	Gonads	5,589	841 (13)	2,913	749 (21)	2,963	498 (14)
13	Gonads	3,634	526 (13)	3,195	849 (21)	4,784	767 (14)
14	Gonads	2,301	369 (14)	1,499	403 (21)	3,321	761 (19)

Note.—The numbers of MBG, FBG, and unbiased genes (UBG) on the autosomes (A) and the X chromosome (X) are shown. Within each expression class (MBG, FBG, UBG), the percentage of genes that is X-linked is given in parentheses.

Given that the head samples include brain tissue and show a much weaker enrichment of MBG on the X chromosome than the brain, it is possible that the signal observed in the head comes primarily from the brain. To investigate this, we looked at the overlap of MBG in head and brain. Overall, the overlap of MBG among the head data sets and the brain is low and only five genes are male-biased in all five head data sets and the brain. However, there is a significant excess of overlapping autosomal MBG between the brain and head for two of the head RNA-seq data sets (data sets 2 and 4) and a significant excess of overlapping X-linked MBG between the brain and head for two of the head RNA-seq data sets (data sets 2 and 3) (supplementary fig. S1, Supplementary Material online). There are 34 genes that are male-biased in the brain and at least two of the five head data sets. Of these, 24 are located on the X chromosome. If the overlapping MBG found in the brain are removed from each head data set, the percentage of X-linked MBG decreases in all cases and only one head data set (data set 4) continues to show a significant excess of X-linked MBG, with 18.2% of the MBG being on the X chromosome (Fisher exact test, *P* < 0.01). Thus, it appears that gene expression in the brain can explain much of the overrepresentation of MBG on the X chromosome that is observed in whole head samples.

### Proximity of Sex-Biased Genes to DCC Binding Sites

**Fig. 2.—:**
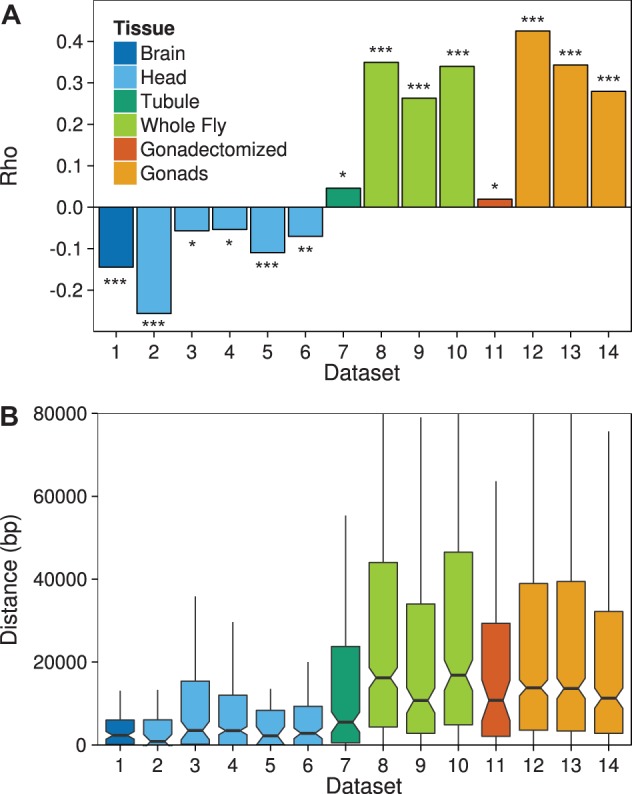
Relationship between sex-biased expression and distance to the nearest DCC binding site. (*A*) The Spearman rank correlation coefficient (Rho) for the correlation between log_2_(male expression/female expression) and distance to the nearest DCC binding site for all X-linked genes in the data sets listed in table 1. *P < 0.05, **P < 0.01, ***P < 0.001. (*B*) Boxplots of the distance between all X-linked MBG and the nearest DCC binding site for each data set.

Because the mechanism of dosage compensation may influence the genomic distribution of sex-biased genes, we examined the correlation between the male/female expression ratio of each gene and its distance to the nearest DCC binding site, as determined by a ChIP-seq experiment using the DCC component maleless (MLE) (Straub et al. 2013). In the brain and in all five head data sets, we observe a significantly negative correlation (fig. 2*A*), indicating that MBG tend to be relatively close to DCC binding sites. In contrast, all of the other data sets show a positive correlation between the male/female expression ratio and distance to the nearest DCC binding site (fig. 2*A*), indicating that MBG tend to be far from DCC binding sites. This is further illustrated in figure 2*B*, which shows that the median distance between an MBG and the nearest DCC binding site is much less for brain and head (ranging from 0.8 to 3.5 kb) than for the other data sets (ranging from 5.5 to 16.8 kb). In the brain and four of the five head data sets (data sets 2, 4, 5, and 6), MBG are significantly closer to DCC binding sites than unbiased genes (Wilcoxon test, *P* < 0.002), whereas for most of the other data sets (data sets 8–12, 14), MBG were significantly further away from DCC binding sites than unbiased genes (Wilcoxon test, *P* < 0.05). For the Malpighian tubule (data set 7) and one of the gonad data sets (data set 13), there was no significant difference between MBG and unbiased genes in their distance to the nearest DCC binding site (Wilcoxon test, *P* > 0.60).

The above results are robust to the DCC component that is used to determine binding site locations, as they also hold for the male-specific-lethal (MSL) proteins MSL-1, MSL-2, and MSL-3 (supplementary fig. S2, Supplementary Material online) (Alekseyenko et al. 2006; Straub et al. 2013). The results also hold for high-affinity sites (HAS), which represent the entry point for DCC binding on the X chromosome and are defined by the colocalization of MLE and MSL-2 (supplementary fig. S2, Supplementary Material online) (Straub et al. 2013).

### Degree of Sex-Biased Expression

**Fig. 3.—:**
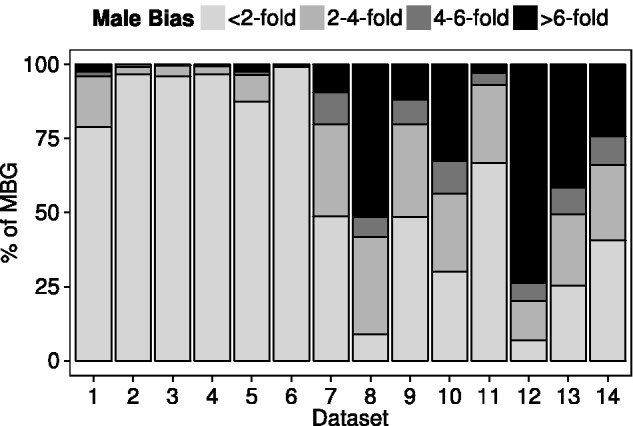
Degree of male-biased expression for the MBG in each data set (table 1). The *Y* axis shows the percentage of MBG that fall into each expression category.

In the above analyses, all MBG were placed in one category, regardless of the extent of their male-biased expression. However, within the MBG there are some striking differences among tissues in the degree of male-biased expression. For the brain and head data sets, only a small proportion of genes (5–20%) show more than a 2-fold male bias in their expression. For all of the other data sets, this proportion is higher, ranging from 30% to 90% (fig. 3). Similarly, the proportion of genes with greater than 4- or 6-fold male bias is less in the brain and head than in all other tissues (fig. 3).

**Fig. 4.—:**
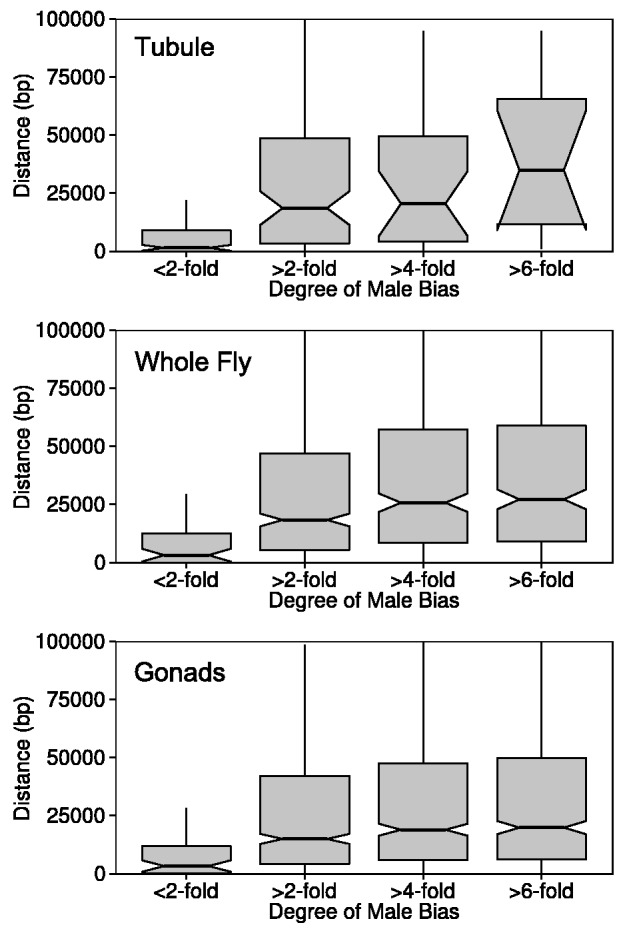
Relationship between the degree of male-biased expression and the distance to the nearest DCC binding site. The data are from RNA-seq data sets 7, 8, and 12 (table 1).

For the RNA-seq data sets that had a sufficient number of highly MBG, we examined the relationship between the degree of male bias and the distance to the nearest DCC binding site. This revealed that genes with weakly male-biased expression tend to be close to DCC binding sites, whereas those with strongly male-biased expression tend to be further away (fig. 4). This pattern held for Malpighian tubule, whole fly, and gonads (fig. 4).

### Expression Level of DCC Components

**Fig. 5.—:**
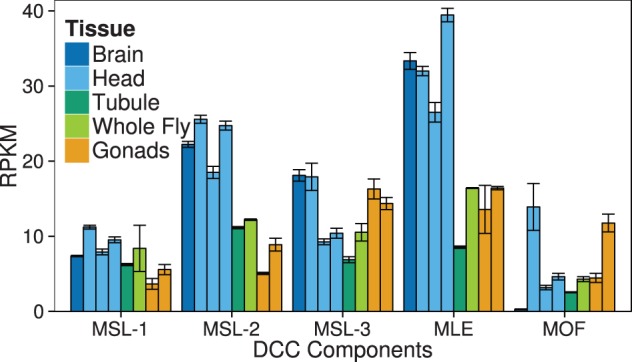
Expression level of DCC components in RNA-seq data sets. Expression level was measured in terms of RPKM. The data are from data sets 1, 2, 3, 4, 7, 8, 12, and 13 (table 1). Error bars indicate the standard error of the mean. MLE and MSL-2 are important for initial recognition of DCC binding sites and their colocalization defines the HAS, whereas MSL-1 and MOF do not colocalize with the other DCC components and are not specific to the X chromosome (Straub et al. 2013).

To investigate possible differences in the level of dosage compensation among tissues, we compared the expression of the five DCC components among all tissues for which RNA-seq data were available. For three DCC components (MLE, MSL-2, and MSL-3), we observe the highest expression in the brain and head (fig. 5). This is especially true for MLE and MSL-2, which colocalize to the HAS at which dosage compensation is initiated (Straub et al. 2013). For both MLE and MSL-2, the expression level in brain and head is approximately 2-fold higher than that in other tissues (fig. 5). This suggests that gene expression in the brain and head may be particularly sensitive to DCC-induced upregulation.

### Gene Age and Expression Breadth

Previous studies found that new genes emerge preferentially on the X chromosome and tend to be both male-biased and tissue-specific (Betrán et al. 2002; Levine et al. 2006; Zhang et al. 2010; Palmieri et al. 2014). Thus, the strong enrichment of X-linked MBG seen in the brain could be explained by these genes being of a relatively young age. To test this possibility, we classified the age of each gene by the point of its first appearance in the *Drosophila* phylogeny (Zhang et al. 2010; Gao et al. 2014). We found no evidence for an enrichment of young MBG on the X-chromosome in the brain. Instead, nearly all of the genes with male-biased expression in the brain were of the oldest age class, being present in all 12 *Drosophila* species (table 3).

**Table 3:** Gene Age, Expression Breadth, and Tissue-Specificity of X-Linked MBG

Data Set	Source	Mean Age^a^	Mean τ^b^	τ > 0.7^c^
1	Brain	5.80	0.36	6 (7%)
2	Head	5.64	0.33	2 (8%)
3	Head	5.81	0.40	7 (7%)
4	Head	5.97	0.36	21 (7%)
5	Head	5.57	0.43	3 (9%)
6	Head	5.90	0.33	4 (3%)
7	Tubule	5.73	0.49	62 (31%)
8	Whole fly	5.22	0.62	216 (41%)
9	Whole fly	5.62	0.58	93 (38%)
10	Whole fly	5.18	0.66	168 (55%)
11	Gonadectomized	5.85	0.52	23 (28%)
12	Gonads	5.38	0.54	237 (33%)
13	Gonads	5.10	0.57	181 (40%)
14	Gonads	5.59	0.56	126 (37%)

^a^Age ranges from 0 (found only in *D*. *melanogaster*) to 6 (found in 12 species of the *Drosophila* genus).

^b^Breadth of expression ranges from 0 (ubiquitously expressed) to 1 (tissue specific).

^c^Number (and percentage) of genes that show high tissue specificity.

To compare the expression breadth of X-linked MBG among data sets, we calculated the statistic τ as a measure of tissue specificity (Yanai et al. 2005; Larracuente et al. 2008). Low values of τ (<0.4) are typical for housekeeping genes, whereas high values (>0.7) indicate high tissue specificity. Overall, genes with male-biased expression in the brain showed very low tissue specificity, with average values of τ falling in the range of housekeeping genes (table 3). Average τ values for X-linked MBG in the brain were similar to those in head, but lower than those in other tissues (table 3). Furthermore, only a small proportion (6.7%) of the X-linked MBG in brain had τ > 0.7 (table 3), and none of these genes had its highest expression signal in the brain. In other words, these genes were male-biased in the brain, but showed highly enriched expression in a different tissue. Thus, there is little evidence for brain-specific function or regulation in this set of genes.

We find that MBG are more tissue-specific than FBG in data sets 7–14 (supplementary fig. S3, Supplementary Material online), which is consistent with a general pattern that has been reported in other studies (Parisi et al. 2004; Meisel 2011; Assis et al. 2012; Meiklejohn and Presgraves 2012). In contrast, in the brain and head (data sets 1–6) there is not a large difference in τ between MBG and FBG (supplementary fig. S3, Supplementary Material online). For four of the head data sets (data sets 3–6), MBG are only slightly, but significantly, more tissue-specific than FBG (Wilcoxon test, *P* < 0.05). However, for the brain and head data set 2, MBG are significantly less tissue-specific than FBG (Wilcoxon test, *P* < 0.05).

## Discussion

Although only 16% of the genes in the *D*. *melanogaster* genome are X-linked, over 75% of the MBG in the brain are located on the X chromosome, which represents a highly significant enrichment. Such a strong enrichment is unique to the brain, although a weaker enrichment is seen in whole head samples, with around 20% of MBG being X-linked (table 2). The opposite pattern is observed in other somatic tissues, gonads and whole flies, where there is a paucity of MBG on the X chromosome (fig. 1*A*). These observations suggest that there are regulatory and/or selective mechanisms that differ between the brain (and to a lesser extent the whole head) and other tissues.

One evolutionary mechanism that is often put forth as an explanation for differences in sex-biased gene content between the X chromosome and the autosomes is sexual antagonism. However, sexual antagonism seems unlikely to explain our observations. Assuming that genes with sex-biased expression serve as a proxy for genes that harbor (or previously harbored) sexually antagonistic variation, the observed patterns could be explained only if intersexual conflict is limited to the brain or if fundamental properties of sexually antagonistic alleles, such as their dominance, differ between the brain and other tissues. Although its role in perception and behavior might suggest that the brain is particularly prone to sexual antagonism, this is not supported by sex-biased gene expression, as the overall number of sex-biased genes and their degree of sex-biased expression are very low in the brain relative to other tissues (table 2, fig. 3). This is not a result of a lack of power to detect sex-biased expression in the brain. The study of the Malpighian tubule, which used the same fly strains, experimental design, and replication scheme, had similar statistical power and uncovered over eight times as many sex-biased genes (table 2) (Huylmans and Parsch 2014). Furthermore, genes showed a much greater degree of sex bias in the tubule than in the brain (fig. 3). Finally, the genes with male-biased expression in the brain do not appear to have brain-specific functions or expression (table 3). Instead, they are mainly housekeeping genes (τ < 0.4) expressed in many tissues or, if they do show narrow expression (τ > 0.7), their tissue of highest expression is not the brain. Thus, they are unlikely candidates to be involved in brain-specific sexual antagonism.

The enrichment of X-linked MBG in the brain also does not appear to be caused by some unusual property of this group of genes. As mentioned above, the genes with male-biased expression in brain tend to be housekeeping genes that have higher expression outside of the brain. However, in most cases, their male-biased expression is observed only in the brain. For example, only 19.5% (25 of 128) of the genes showing male-biased expression in the brain also show male-biased expression in a whole-fly meta-analysis (data set 10). Thus, genes that are male-biased in the brain tend to be globally expressed, but not globally male-biased. In addition, the MBG in the brain are not of an unusually young evolutionary age (table 3). It has been shown that young genes may preferentially arise on the X chromosome and be male-biased in their expression (Betrán et al. 2002; Levine et al. 2006; Zhang et al. 2010; Palmieri et al. 2014). However, almost all of the X-linked MBG in the brain are of the oldest age class and are present in the genomes of species from across the *Drosophila* genus.

Our finding that the ratio of male/female expression is significantly correlated with the distance to the nearest DCC binding site in all 14 data sets suggests that the mechanism of dosage compensation plays a role in determining sex-biased expression. Interestingly, the correlation is negative for the brain and the head, but positive for all other tissues (fig. 2*A*). Consistent with this, MBG in the brain and head tend to be much closer to DCC binding sites than those in other tissues (fig. 2*B*). The observed correlations are unlikely to be spurious, as they are, in most cases, highly significant and they hold for binding sites of several different DCC components that were detected in independent experiments (supplementary fig. S2, Supplementary Material online). We propose that the interplay between dosage compensation and sex-specific gene regulation can explain our observations and the inconsistencies between previous studies (Bachtrog et al. 2010; Chang et al. 2011; Catalán et al. 2012). For genes with sex-specific regulation, particularly those that require high expression in males, the binding of the DCC and its associated chromatin modification could interfere with the binding of transcription factors that positively regulate male expression and prevent further upregulation in males. Thus, strongly MBG would be expected to be located far away from DCC binding sites. For genes lacking sex-specific regulation, being in close proximity to a DCC binding site might result in overcompensation, whereby a gene’s transcription is increased by more than 2-fold, resulting in male-biased expression. In this case, the resulting degree of male bias is expected to be rather weak, as it depends on dosage compensation overshooting its target of 2-fold hypertranscription of the male X chromosome.

A more consistent pattern across the different tissues emerges if we assume that genes showing more than a 2-fold male bias in their expression are controlled by their own individual sex-specific regulatory factors, whereas those showing less than a 2-fold male bias generally lack sex-specific regulatory elements. In the brain and head, where the vast majority of MBG show only weak male bias (fig. 3), the MBG tend to be close to DCC binding sites and their male-biased expression may result mainly from an overshoot in dosage compensation. This could explain the overabundance of MBG on the X chromosome and the observed negative correlation between the ratio of male/female expression and distance to the nearest DCC binding site. In other tissues, a much higher proportion of genes show highly male-biased expression (fig. 3) and these genes tend to be located far away from DCC binding sites (fig. 4). As these highly MBG have a large effect on the correlation between the ratio of male/female expression and distance to the nearest DCC binding site, an overall positive correlation is seen. It should be noted, however, that even when genes with greater than 2-fold sex-bias (male and female) are excluded, significant (albeit weaker) correlations between male/female expression and distance to the nearest DCC binding site are still observed in many data sets (supplementary fig. S4, Supplementary Material online). In these cases, it is mainly the FBG that drive the correlation: FBG show the opposite pattern as MBG with regard to DCC distance, but the distribution of DCC distances for FBG is not affected by removal of strongly sex-biased genes to the same extent that it is for MBG (supplementary fig. S4, Supplementary Material online).

Recently it has been reported that the observed positive correlation between male-biased expression and distance to the nearest DCC binding site in gonadectomized flies is driven by genes with highly testis-enriched expression and that the correlation is no longer significant when these genes are excluded (Vensko and Stone 2014). This suggests that the influence of dosage compensation on male-biased gene expression is not organism-wide. Our results support this interpretation, but further suggest that the influence of dosage compensation on male-biased gene expression varies among somatic tissues (fig. 2*A*). It is possible that the pattern reported for gonadectomized flies stems either from contamination with gonadal tissue or from testis-biased genes having sufficient residual expression in somatic tissues to be detected in gonadectomized flies (Vensko and Stone 2014). Contamination with gonadal tissue is very unlikely to affect the results for brains or heads, as they can be separated cleanly from gonads during dissection. It is also unlikely that the observed patterns in these tissues are driven by residual expression of testis-biased genes, as there tends to be very little overlap among the genes that are male-biased in testis and those that are male-biased in brain or head (supplementary fig. S1, Supplementary Material online). However, we did find that a large proportion of genes with male-biased expression in the brain have female-biased expression in the gonads (supplementary file S1, Supplementary Material online). Of the 97 X-linked MBG in brain, 59 were FBG in at least one of the gonad data sets (and not MBG in any gonad data set). This overlap is consistent with the contrasting patterns we see for the two tissues: In the brain MBG are enriched on the X chromosome and close to DCC binding sites, whereas in the gonad FBG are enriched on the X chromosome and close to DCC binding sites. Thus, it is possible that the gonadal expression of these genes could partly explain the patterns seen in the brain. However, the enrichment of FBG on the X chromosome in gonad is not nearly as strong as the enrichment of MBG on the X chromosome in brain (fig. 1). Furthermore, if we exclude all genes with female-biased expression in gonad from our analysis, we still detect a strong and highly significant enrichment of MBG on the X chromosome and a significantly negative correlation between male/female expression and DCC distance in the brain (supplementary fig. S5, Supplementary Material online). Thus, gonadal expression alone cannot explain the patterns observed in the brain.

The overcompensation of X-linked genes in the male brain might be enhanced relative to other tissues, if the brain is more sensitive to dosage compensation. The two major components of the DCC that bind to the X chromosome to initiate dosage compensation, MLE and MSL-2 (Straub et al. 2013), show exceptionally high expression in the brain (fig. 5). A similar result has been reported by Vensko and Stone (2015), who found that the brain had the highest expression of the *msl-2* gene among all adult tissues included in FlyAtlas (Chintapalli et al. 2007). If the abundance of MLE and MSL-2 is indicative of the level of DCC binding and dosage compensation in a tissue (Dahlsveen et al. 2006), then one would expect the brain to be enriched with genes that overshoot the expected 2-fold upregulation. The sensitivity of the brain (and head) to dosage compensation is further suggested by a recent study in *Drosophila pseudoobscura* that found dosage compensation of the newly evolved neo-X chromosome to be incomplete in whole flies, but complete in the head (Nozawa et al. 2013). If neo-sex chromosomes achieve dosage compensation by recruiting the DCC machinery, which appears to be the case in *Drosophila* (Ellison and Bachtrog 2013), then tissues such as the brain and head, which have high expression of MLE and MSL-2, may be the first to show complete dosage compensation. At present, it is not known whether the rapid establishment of complete dosage compensation in the brain/head is favored by natural selection, or whether it is a neutral side effect of having high MLE and MSL-2 expression in these tissues.

**Fig. 6.—:**
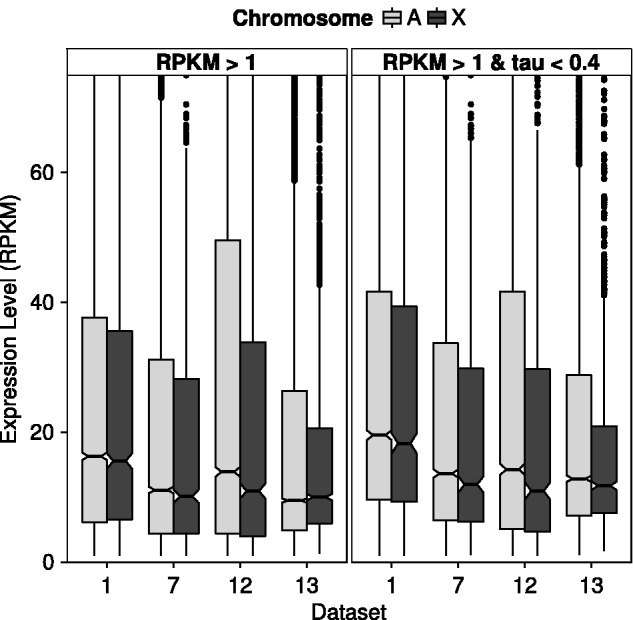
Expression level of autosomal and X-linked genes in males. Data sets 1 and 7 correspond to the brain and Malpighian tubule, respectively, whereas data sets 12 and 13 correspond to the gonads (table 1). The left panel includes all expressed genes (RPKM > 1). Only data set 12 shows a significant difference in expression between X-linked and autosomal genes (Wilcoxon test, *P* < 0.001). The right panel includes only broadly expressed “housekeeping” genes (RPKM > 1, τ < 0.4). There is a significant difference between X-linked and autosomal expression for data set 12 (Wilcoxon test, *P* < 0.001), as well as for data sets 7 and 13 (Wilcoxon test, *P* < 0.05).

There have been conflicting reports as to whether or not dosage compensation occurs in the male germline of *D*. *melanogaster* (Gupta et al. 2006; Deng et al. 2011; Meiklejohn et al. 2011; Meiklejohn and Presgraves 2012). If dosage compensation does occur, it is thought to be through a mechanism that is independent of the DCC (Gupta et al. 2006). Thus, a strong correlation between male-biased expression and distance to the nearest DCC binding site is not necessarily expected in the gonads (figs. 2 and 4). From our analysis of the gonad RNA-seq data sets (data sets 12 and 13), we see some evidence for incomplete dosage compensation, especially when looking at housekeeping genes (fig. 6). However, even for these genes, the ratios of the median expression of the autosomes to the X chromosome are only 1.30 and 1.09, for data sets 12 and 13, respectively. Furthermore, the brain and Malpighian tubule show similar ratios (1.07 and 1.14, respectively) (fig. 6). Thus, it is not clear whether the expression difference between the autosomes and the X chromosome in the gonads reflects an absence of dosage compensation, or whether it reflects a more general pattern of feminization of the X chromosome (fig. 1*B*). If dosage compensation does not occur in the male germline, it could be that the correlation between male-biased expression and DCC distance stems from the same genes having male-biased expression in other tissues where dosage compensation occurs. For example, of the genes showing male-biased expression in the gonads, 16–22% (depending on the data set) are also male-biased in the Malpighian tubule. This however, does not explain why the correlation between sex-biased expression and DCC binding site distance is stronger for the gonads than for other tissues (fig. 2*A*). Another possibility is that, if dosage compensation does not occur in the male germline, then there has been no selective pressure to maintain (or acquire) DCC binding sites in the proximity of gonad-specific MBG. Finally, it could be that an unknown regulatory mechanism, distinct from dosage compensation, also relies on DCC binding sites and/or components of the DCC in the male germline. It has been shown that the expression of X-linked testis-specific genes is globally suppressed in the male germline in a manner that is independent of gene dose (Hense et al. 2007; Kemkemer et al. 2011, 2014). It is possible that this suppression takes advantage of elements of the dosage compensation apparatus that are already in place and are specific to the X chromosome.

If the excess of X-linked MBG in the brain is caused by an overshooting of the expected 2-fold dosage compensation of genes located close to DCC binding sites, then we expect this phenomenon to be limited to species such as *D. melanogaster* that achieve dosage compensation through hypertranscription of the male X chromosome. In female heterogametic species, such as birds, a significant enrichment of MBG on the Z chromosome has been observed (Kaiser and Ellegren 2006; Storchova and Divina 2006). However, this does not appear to be tissue-specific and is likely caused by the absence (or incompleteness) of Z chromosome dosage compensation in females (Ellegren et al. 2007; Itoh et al. 2007). In mammals, dosage compensation is achieved by inactivating one of the X chromosomes in females. In the mouse, sex-biased expression varies considerably among tissues, with a relatively low proportion of sex-biased genes in the brain (Yang et al. 2006; Reinius et al. 2012). The genes with sex-biased expression in the mouse brain tend to show a small degree of sex-biased expression, with an excess of FBG and a paucity of MBG on the X chromosome (Reinius et al. 2012). There also appears to be a core set of X-linked genes that escape dosage compensation over several tissues, including the brain (Reinius et al. 2012). Thus, there are some similarities with the *Drosophila* observations. Although the mechanisms differ greatly between *Drosophila* and mammals, there is evidence that tissue-specific variation in dosage compensation may influence sex-biased gene expression in both taxa.

## Supplementary Material

Supplementary file S1, figures S1–S6, and tables S1 and S2 are available at *Genome Biology and Evolution* online (http://www.gbe.oxfordjournals.org/).

Supplementary Data
