# Supplementary material for: Variation in the X:Autosome Distribution of Male-Biased Genes among Drosophila melanogaster Tissues and Its Relationship with Dosage Compensation
Source: Genome Biol Evol. 2015 Jun 24;7(7):1960–71. doi: 10.1093/gbe/evv117 (PMC4524484; doi:10.1093/gbe/evv117)
Supplement: Supplementary Data [file supp_7_7_1960__index.html]

Variation in the X:Autosome Distribution of Male-Biased Genes among Drosophila melanogaster Tissues and Its Relationship with Dosage Compensation — Supplementary Data 

# Variation in the X:Autosome Distribution of Male-Biased Genes among *Drosophila melanogaster* Tissues and Its Relationship with Dosage Compensation

## Supplementary Data

files

- Supplementary Data - xls file
- Supplementary Data - xls file
- Supplementary Data - xls file
- Supplementary Data - xls file
- Supplementary Data - xls file
- Supplementary Data - pdf file
