# Supplementary material for: Variation in the X:Autosome Distribution of Male-Biased Genes among Drosophila melanogaster Tissues and Its Relationship with Dosage Compensation
Source: Genome Biol Evol. 2015 Jun 24;7(7):1960–71. doi: 10.1093/gbe/evv117 (PMC4524484; doi:10.1093/gbe/evv117)
Supplement: Supplementary Data [file supp_evv117_Supp_Mat.pdf]

Table S1. NCBI SRA accession numbers of raw RNA-seq data

Dataset	Source	Female	Male
1	Brain	SRX187181, SRX187182, SRX187185, SRX187186	SRX187183, SRX187184, SRX187187, SRX187188
2	Head	SRR073273, SRR073277, SRR073278, SRR073279, SRR073280, SRR073281	SRR073282, SRR073283, SRR073284, SRR073285, SRR073286, SRR073287
3	Head	SRR999232, SRR999233, SRR999234, SRR999268, SRR999269, SRR999270	SRR999235, SRR999236, SRR999237, SRR999238, SRR999239, SRR999240, SRR999241
4	Head	SRR039433, SRR039434, SRR039435, SRR039445	SRR039436, SRR039437, SRR039438, SRR039452
7	Tubule	SRX608310, SRX608311, SRX608314, SRX608315	SRX608312, SRX608313, SRX608316, SRX608317
8	Whole fly	SRR166807, SRR166808	SRR166809, SRR166810
12	Gonads	SRR070431, SRR100277, SRR100283	SRR070422, SRR070423, SRR100276, SRR350960. SRR350961
13	Gonads	SRR032310, SRR032311, SRR032312, SRR032313	SRR032304, SRR032305, SRR032306, SRR032307

Table S2. Results using alternative statistical and mapping software

Mapper	Statistics	Filter	MBG_A	MBG_X	*P* (X enrichment)	*P* (DCC distance)
Stampy	edgeR	–	39	53	5.9E-11	2.5E-4
Stampy	baySeq	–	4	22	1.0E-10	5.4E-5
NextGenMap	DESeq2	–	36	111	8.8E-11	3.9E-5
NextGenMap	DESeq2	RPKM > 1	25	86	6.7E-11	7.0E-4
NextGenMap	DESeq2	FDR = 1%	17	67	7.0E-11	5.4E-4

The numbers of male-biased genes (MBG) on the autosomes (A) and X chromosome (X) are given for different combinations of mapping and statistical software. The final two columns give the *P*-values for tests of enrichment of MBG on the X chromosome (Fisher exact test) and for comparisons of distance to DCC binding sites between MBG and unbiased genes (Wilcoxon test). In all cases, MBG were significantly enriched on the X chromosome and were significantly closer to DCC binding sites than unbiased genes. Values shown are for MLE binding sites.

A) Male-biased genes, X chromosome

	Brain (1)	Head (2)	Head (3)	Head (4)	Tubule (7)	Fly (8)	Gonad (12)	Gonad (13)
Brain (1)		0.0141	0.0116	0.1433	0.5202	0.0174	0.0707	0.0068
Head (2)	11 (2)		0.0133	0.0057	0.2601	0.8924	0.4334	0.3125
Head (3)	20 (7)	12 (3)		0.001	0.8171	0.9658	0.7998	0.4973
Head (4)	34 (23)	24 (8)	58 (0)		0.9031	0.1087	1.05E-05	9.34E-06
Tubule (7)	16 (13)	9 (5)	17 (16)	54 (53)		0.003424	0.0559	0.0016
Fly (8)	17 (34)	11 (12)	45 (45)	124 (151)	112 (72)		2.20E-16	2.20E-16
Gonad (12)	30 (46)	14 (18)	57 (60)	122 (201)	126 (97)	530 (259)		2.20E-16
Gonad (13)	11 (28)	7 (11)	31 (37)	63 (124)	99 (59)	390 (158)	453 (231)	

B) Male-biased genes, autosomes

	Brain (1)	Head (2)	Head (3)	Head (4)	Tubule (7)	Fly (8)	Gonad (12)	Gonad (13)
Brain (1)		0.0268	0.2441	0.0152	0.1715	0.2092	0.4813	0.9616
Head (2)	6 (0)		0.0051	5.64E-05	0.2093	0.4491	0.0217	0.0032
Head (3)	6 (3)	25 (9)		4.56E-12	0.0003	0.4069	9.69E-07	1.18E-11
Head (4)	18 (6)	55 (20)	271 (132)		0.0042	0.0159	2.20E-16	6.84E-14
Tubule (7)	9 (4)	9 (15)	53 (97)	173 (231)		3.34E-16	4.75E-09	2.20E-16
Fly (8)	22 (14)	47 (55)	367 (345)	733 (828)	773 (484)		2.20E-16	2.20E-16
Gonad (12)	15 (19)	47 (72)	320 (456)	708 (1094)	870 (642)	3620 (2123)		2.20E-16
Gonad (13)	12 (12)	22 (46)	150 (293)	449 (703)	706 (410)	2588 (1362)	3058 (1813)	

C) Female-biased genes, X chromosome

	Brain (1)	Head (2)	Head (3)	Head (4)	Tubule (7)	Fly (8)	Gonad (12)	Gonad (13)
Brain (1)		0.0249	0.0304	0.0047	0.0628	0.6886	0.5028	0.8179
Head (2)	9 (2)		5.69E-05	0.0003	0.2877	0.0112	0.0024	0.0016
Head (3)	17 (7)	31 (6)		3.10E-06	0.0144	0.0763	0.5597	0.7336
Head (4)	27 (10)	33 (9)	90 (37)		0.0004	0.7367	0.5545	0.3381
Tubule (7)	19 (9)	15 (10)	62 (37.58)	102 (58)		0.0003	0.0119	0.0002
Fly (8)	26 (23)	10 (25)	73 (96)	142 (148)	189 (126)		2.20E-16	2.20E-16
Gonad (12)	18 (22)	7 (24)	84 (92)	131 (141)	163 (121)	588 (288)		2.20E-16
Gonad (13)	23 (25)	8 (27)	97 (112)	174 (157)	200 (133)	640 (319)	612 (306)	

D) Female-biased genes, autosomes

	Brain (1)	Head (2)	Head (3)	Head (4)	Tubule (7)	Fly (8)	Gonad (12)	Gonad (13)
Brain (1)		0.0028	0.0222	0.0098	0.3964	0.0005	0.0194	0.5758
Head (2)	15 (3)		2.21E-13	1.44E-06	0.1731	6.60E-13	4.60E-12	1.07E-06
Head (3)	20 (8)	103 (21)		2.20E-16	0.0109	0.1395	0.0461	0.4228
Head (4)	33 (15)	96 (40)	306 (128)		2.12E-08	0.9682	0.9605	0.0005
Tubule (7)	9 (6)	26 (17)	83 (53)	200 (103)		4.12E-08	5.81E-06	3.56E-11
Fly (8)	9 (31)	18 (94)	256 (291)	570 (569)	311 (188)		2.20E-16	2.20E-16
Gonad (12)	13 (28)	15 (84)	216 (259)	505 (507)	263 (169)	2187 (855)		2.20E-16
Gonad (13)	26 (30)	36 (91)	300 (281)	672 (550)	332 (182)	2237 (925)	2086 (831)	

Fig. S1. Overlap of sex-biased genes among RNA-seq datasets. For each category the observed overlap is shown below the diagonal. The expected number of overlapping genes is given in parentheses. A significant excess of overlaps is indicated by dark green shading, while a significant paucity of overlaps is indicated by light green shading. The *P*-value of the overlap (goodness of fit test) is given above the diagonal, with significant values shaded red.

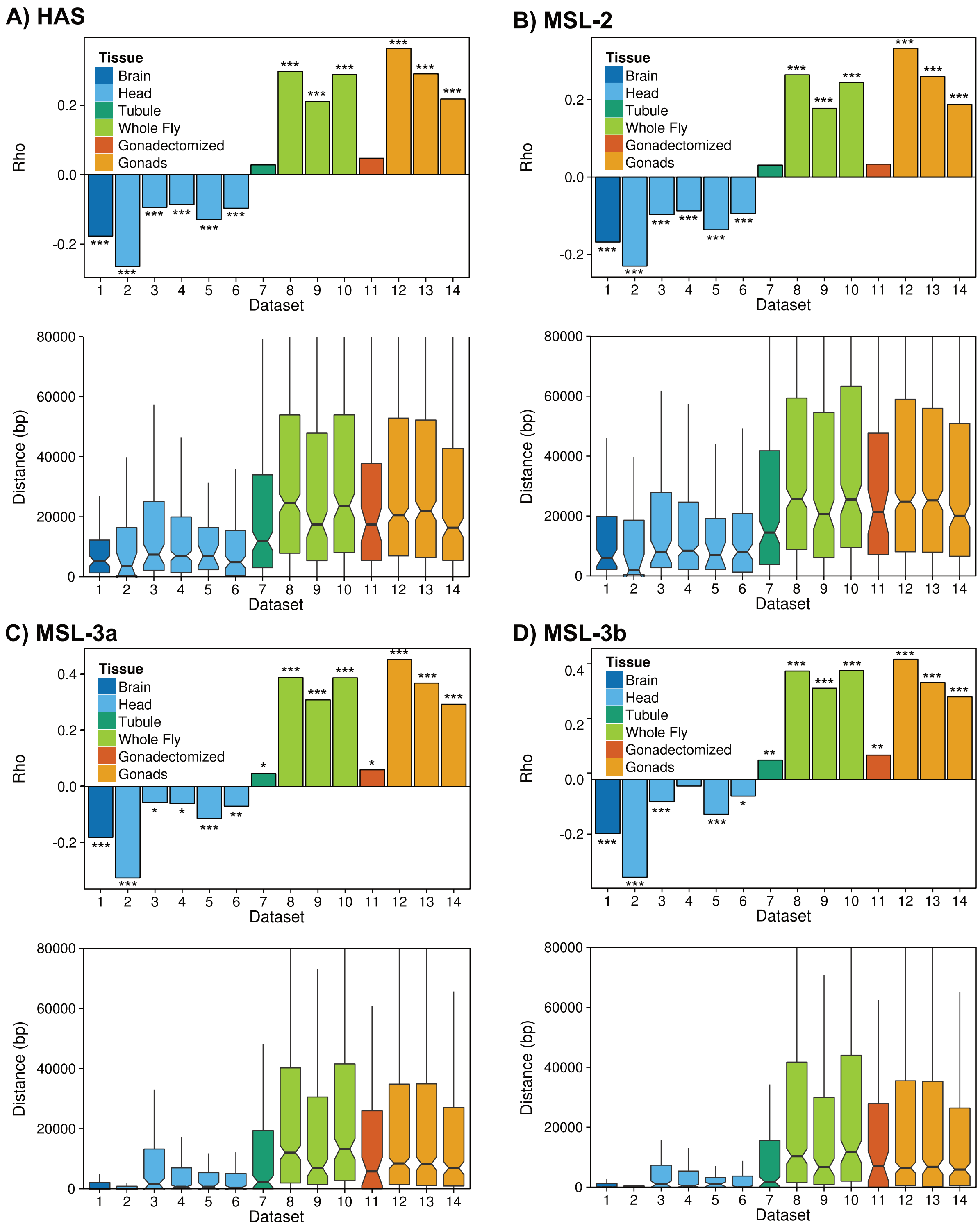

Fig. S2. Spearman rank correlation between $\log_2$(male expression/female expression) and distance to the nearest DCC binding site, and the distribution of the distances of MBG to the nearest DCC binding site as determined for different DCC components (A–D). HAS and MSL-2 data are from Straub et al. (2013). Data from two different MSL-3 ChIP experiments are shown: MSL-3a represents ChIP-seq data from Straub et al. (2013), while MSL-3b represents ChIP-chip data from Alekseyenko et al. (2006).

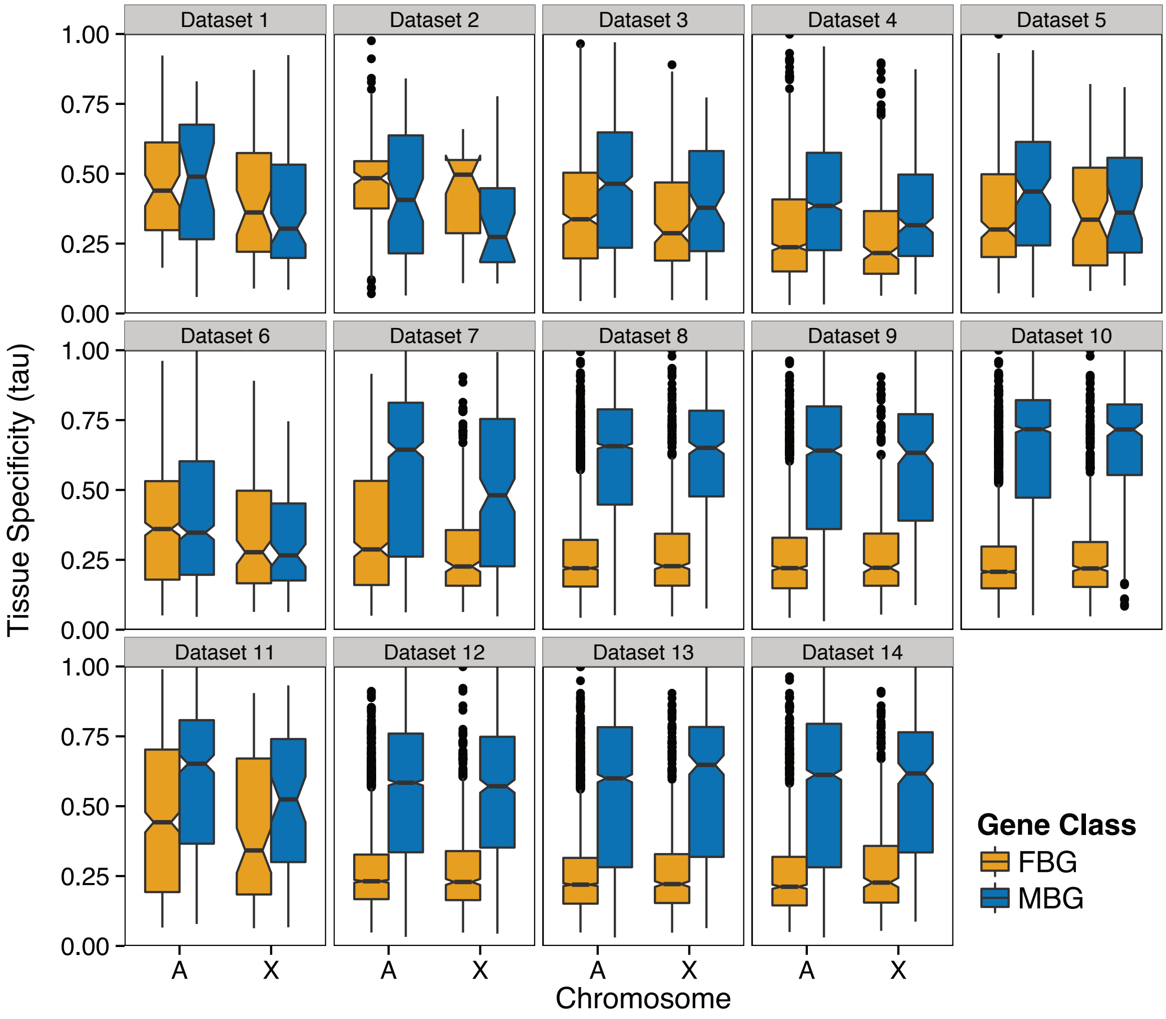

Fig. S3. Measure of tissue specificity (tau) for autosomal and X-linked female-biased and male-biased genes in each dataset. Datasets correspond to those in table 1.

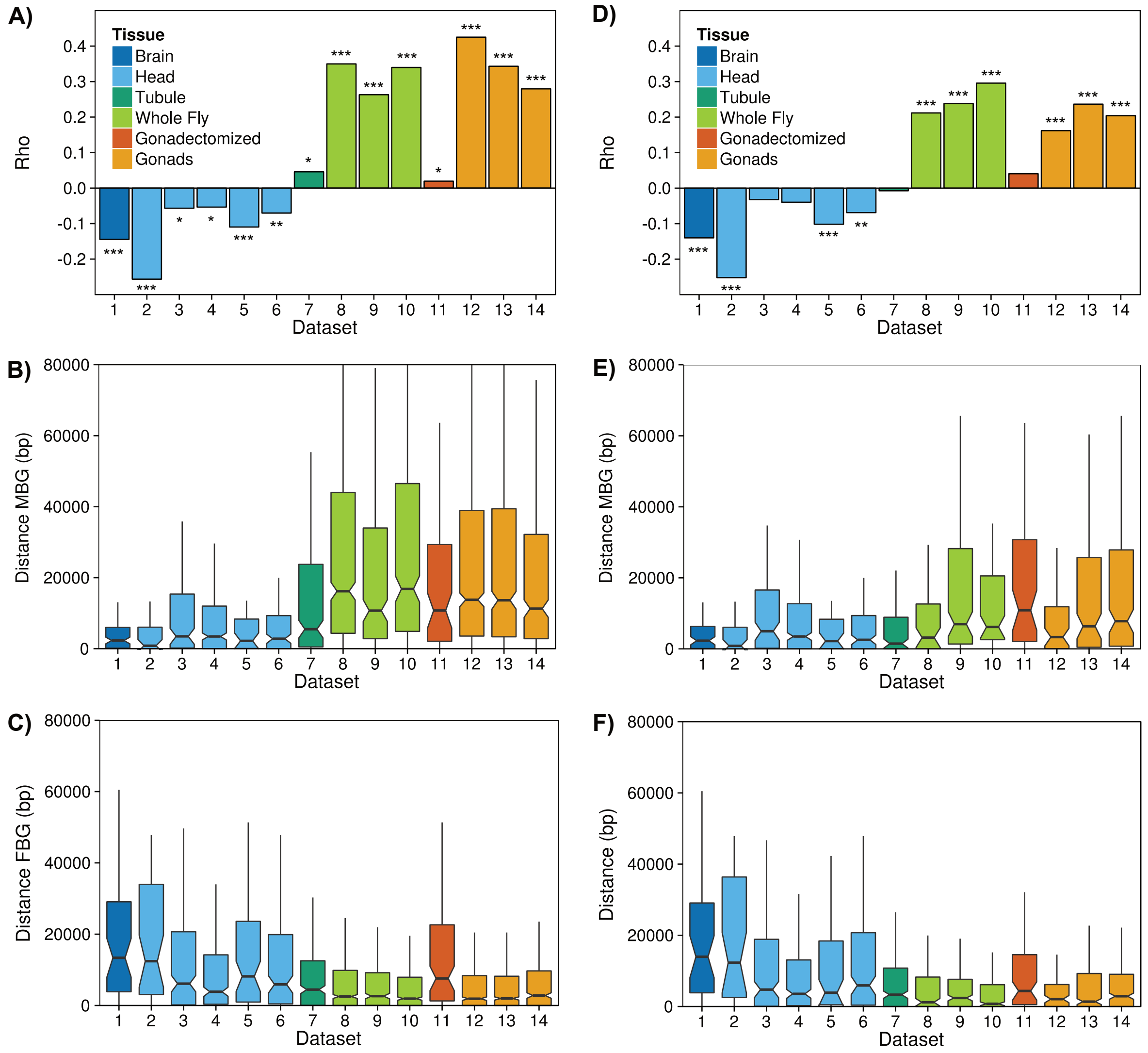

Fig. S4. Relationship between sex-biased expression and distance to the nearest DCC binding site. Panels A–C are for all genes. Panels D–F are for all genes after excluding those with >two-fold sex-bias in either sex.

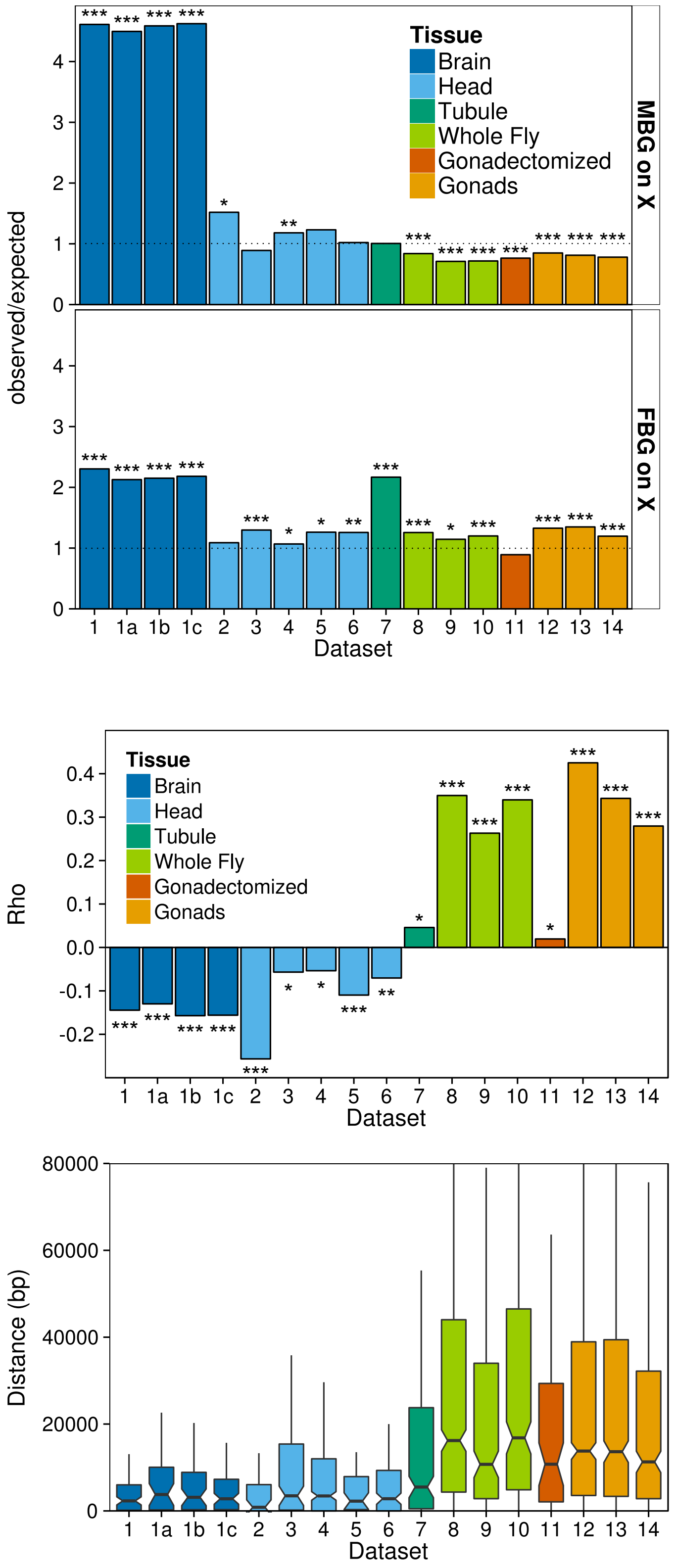

Fig. S5. Relative abundance of sex-biased genes on the X chromosome (top panel) and relationship between sex-biased expression and distance to the nearest DCC binding site (bottom panel). The figures are the same and figs. 1 and 2 of the main manuscript, but with the addition of brain datasets 1a, 1b, and 1c, in which genes with female-biased expression in ≥1, ≥2, or 3 gonad datasets were excluded, respectively. $*P < 0.05$, $**P < 0.01$, $***P < 0.001$.

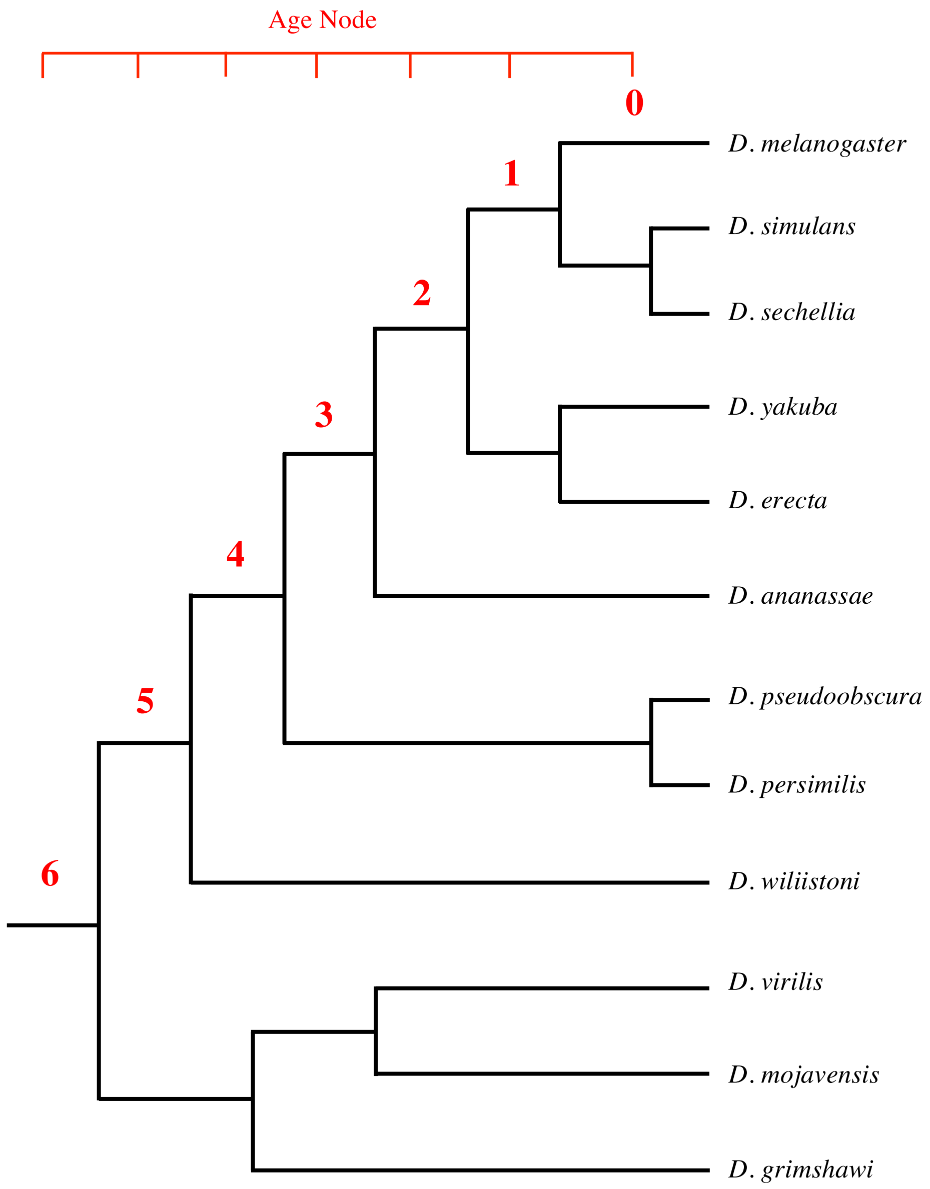

Fig. S6. Method to determine gene age. A gene's age class is determined from the branch on which it first appears in the *Drosophila* phylogeny. For example, a gene that is found only in *D. melanogaster* is given an age of zero, while a gene present in all 12 species is given an age of six.
